# The succession pattern of soil microbial communities and its relationship with tobacco bacterial wilt

**DOI:** 10.1186/s12866-016-0845-x

**Published:** 2016-10-06

**Authors:** Jiaojiao Niu, Zhongwen Rang, Chao Zhang, Wu Chen, Feng Tian, Huaqun Yin, Linjian Dai

**Affiliations:** 1 School of Minerals Processing and Bioengineering, Central South University, Changsha, 410083 China; 2 Key laboratory of Biometallurgy, Ministry of Education, Changsha, 410083 China; 3 College of agronomy, Hunan Agricultural University, Changsha, 410128 China; 4 Tobacco monopoly bureau of Xiangxi Autonomous Prefecture, Hunan Jishou, 416000 China

**Keywords:** Soil microbial communities, Succession mechanism, Crop health, Illumina sequencing, Molecular ecological network

## Abstract

**Background:**

The interaction mechanism between crop and soil microbial communities is a key issue in both agriculture and soil ecology. However, how soil microbial communities respond to crop planting and ultimately affect crop health still remain unclear. In this research, we explored how soil microbial communities shifted during tobacco cultivation under different rotation systems (control, maize rotation, lily rotation and turnip rotation).

**Results:**

Our analyses showed that soil microbial communities had a general response pattern to tobacco planting, as the abundances of *Proteobacteria* and *Planctomycetes* increased while *Acidobacteria* and *Verrucomicrobia* decreased during tobacco cultivation, no matter which rotation system was adopted. Notably, tobacco decreased the diversity and co-occurrence of soil microorganisms, but maize rotation might suppress tobacco bacterial wilt by alleviating the decrease in biodiversity and co-occurrence. Molecular ecological network analysis indicated that there was stronger competition between potential disease suppressive (e.g., *Acidobacteria*) and inducible bacteria (e.g., *Chloroflexi*) in maize rotation systems. Both soil properties (e.g., pH, Ca content) and microbial communities of tobacco mature period depended on their counterparts of fallow period, and all these factors shaped tobacco disease comprehensively.

**Conclusions:**

Both soil microbial communities of fallow stage and tobacco selection shaped the communities of tobacco mature stage. And effective rotation crop (maize) could decrease the incidence of tobacco bacterial wilt by alleviating the decrease in diversity and co-occurrences of microbial populations. This study would deepen our understanding about succession mechanism of soil microbial communities during crop cultivation and their relationship with crop health.

**Electronic supplementary material:**

The online version of this article (doi:10.1186/s12866-016-0845-x) contains supplementary material, which is available to authorized users.

## Background

Soil microbes are drivers of plant diversity and productivity in terrestrial ecosystems, while plants also affect soil physical and chemical environment as well as soil organisms. Unrevealing how soil microbial communities change in response to crop planting is a central issue in both ecology and agriculture [1]. Particularly, how pathogen populations response to crop planting is significant for exploring pathogenesis of crop disease. It has been suggested that disease inducible microorganisms originated from initial soil microbial communities before crop planting [2]. Researches in this area make it possible to control pathogen populations before they cause severe crop disease [3, 4], but few studies explore the relationship between succession of soil microbial communities and plant health.

However, the response of microbial communities to crop planting is often unpredictable and too variable. It is difficult to summarize a general response pattern, because a kind of crop (e.g., wheat, tobacco) could be planted in farmlands with different climates, soil properties and rotation systems, resulting in spatial and temporal variability of soil microbial communities. Some DNA-based studies indicated the importance of spatial variability in microbial communities [5, 6]. Meanwhile, other studies demonstrated the significance of temporal variability, so examining the effect of management practices on soil quality based on microbial communities should consider seasonal changes [7]. Due to these variations, studies about soil microbial communities of a same crop were usually not complied with each other. Take soil microbial communities of tobacco as an example, it was reported in a research that fungi and actinomycete had similar abundance pattern, and both of them descended at the early stages then ascended [8], while in another study fungi reached peak at rapid growth stage and the largest number of actinomycete appeared in rosette stage [9]. In a word, the spatial and temporal variabilities of microbial communities are ubiquitous, therefore such background noises must be excluded, at least taken into consideration, when exploring general response pattern of soil microbial communities to crop planting.

Analytical method is another limitation in investigating soil microbial communities. It has been known that interactions among microbial populations are ubiquitous [10], which provide a better understanding of the relationship between complexity and ecological stability. However, few studies involved interactions among microorganisms, except for several researches of plants pathogens and their antagonistic bacteria [11, 12]. Mainly based on experiments, these studies provided little information about complex interactions among different microbial populations, which might affect each other directly or indirectly. Random matrix theory (RMT) is powerful in identifying molecular ecological networks in microbial communities, and it has been employed well to analyze the co-occurrence/interaction among different microbial populations [13, 14]. It provides us an opportunity to explore how co-occurrences/interactions among different microbial populations change with plant growth and disease occurrence, which is an issue poorly studied in agriculture.

Tobacco is a typical continuous cropping intolerant crop, for continuous cropping usually induces serious crop disease, such as tobacco bacterial wilt and black shank [15, 16]. Thus it usually be cultivated under different rotation systems (e.g., turnip, wheat, rice and maize). Studies about function mechanism of crop rotation have revealed that it is related to soil microbes, but how microbial communities shift after rotation crop planting and ultimately shape tobacco health still remain unclear [16, 17]. These characteristics make tobacco a suitable subject to explore succession of soil microbial communities, especially pathogens, during crop planting. Tobacco has 4 important growth stages, including transplanting stage, rosette stage, fast-growing stage and mature stage. Mature stage is also the disease stage when soil microorganisms, at least pathogens, are most active. In order to reveal the response mechanism of soil microorganisms to tobacco planting and disease, we chose two key time points: the time before tobacco transplanting and the time during tobacco disease period. We hypothesized that (i) soil microbial communities had a general succession pattern from fallow period to tobacco mature period; (ii) rotation crops affected tobacco health through influencing soil microbial communities and their succession. Using 16S rRNA gene sequencing, we explored the general effects of tobacco planting on microbial community composition, structure and co-occurrence pattern across 4 different rotation types. As a result, we revealed the succession pattern of soil microbial communities during tobacco plating, and found that maize rotation could suppress tobacco bacterial wilt by alleviating the decrease in biodiversity and co-occurrences among bacterial populations.

## Methods

### Sampling, Illumina sequencing and data processing

Tobacco cultivated under different rotation systems was investigated, which were continuous tobacco cropping (Control), tobacco-maize rotation cropping (MR), tobacco-lily rotation cropping (LR), and tobacco-turnip rotation cropping (TR). Samples were collected from each field using checkerboard sampling method on March 27th (fallow period before tobacco planting) and July 28th (tobacco mature stage or diseased period) respectively. Field design, sampling, sample processing, measurement of soil properties, DNA extraction and 16S rRNA gene sequencing were conducted following the methods described before [18]. All the 16S rRNA sequences were deposited in GenBank database and the accession numbers were KR831285 - KR855564. Dissimilarity tests were based on Bray-Curtis dissimilarity index using analysis of similarities (ANOSIM) [19]. Differences in abundances among 4 groups were determined by a one-way analysis of variance (ANOVA) followed by least significant difference (LSD) test [20]. Bacterial community diversity was calculated using Shannon-Weiner’s H′ and evenness. Multivariate statistical analyses of sequencing data were conducted, including detrended correspondence analysis (DCA) for comparing the different microbial communities, as well as mantel test [21] and partial least squares path modeling (PLSPM) for linking microbial communities to environmental variables. All the analyses were performed in R v. 2.6.1 with the packages *vegan* and *plspm* [22] or online (http://ieg.ou.edu/).

### Network construction and characterization

As previously described, random matrix theory (RMT)-based approaches were used for network construction [14, 23], hub and connector gene identification, and topological property determination with an automatic threshold. To ensure correlation reliability, OTUs in at least 5 out of 8 replicates were used for network analysis. Various network properties such as average degree, average path distance, average clustering coefficient and modularity index were characterized. The network modules were generated using rapid greedy modularity optimization.

The experimental data used for constructing phylogenetic molecular ecological networks (pMEN) were based on 16S rRNA gene sequencing analysis. First, a Pearson correlation matrix was constructed [24]. The correlation matrix was then converted to a similarity matrix, which measures the degree of concordance between the abundance profiles of OTUs across different samples by taking the absolute values of the correlation matrix [24, 25]. Subsequently, an adjacency matrix, which encodes the connection strength between each pair of nodes, was derived from the similarity matrix by applying an appropriate threshold, which was defined using the RMT-based network approach as previously described [23, 26, 27]. The Cytoscape 2.6.0 [28] software was used to visualize the network graphs. Other information about genes (e.g., taxonomy, relative abundance) and edge information (e.g., weights and positive and negative correlations) was also imported into the software and visualized in the network figures. Since we are interested in the temporal variability of network interactions, the pMENs were constructed separately based on sequencing data of Control, MR, LR and TR of 2 periods, respectively.

## Results

### Soil geochemical properties

A summary of soil properties, including soil pH, water content and amount of Ca, K, Mn, Fe, Co, Cr and Ni, was described in Additional file 1: Table S1. Water content was significantly (*p* < 0.01) higher in fallow period (*T*-test) and showed no difference among 4 rotation groups. The value of pH ranged from 4.43 to 5.40, remained unchanged in MR and TR, but significantly (*p* < 0.05) higher in Control and lower in LR. But it showed no significant difference between 2 periods. ICP analysis revealed the total amount of various elements. Most of them were more abundant in fallow period (e.g. Fe and Cr) except for K, which were significantly (*p* < 0.05) higher in tobacco mature period. The amount of elements also showed difference among four rotation systems. For example, K and Ni were significantly (*p* < 0.05) less abundant in Control, as well as the amount of Ca was higher in MR. Besides, Control and TR had higher tobacco disease rate (57.78 % and 59.62 %), while it was the lowest in MR (23.54 %).

### Overview of microbial community diversity

After resample, we obtained 18000 high-quality 16S rRNA gene sequences per sample. Rarefaction curve showed that the numbers of OTUs were almost saturate in all samples, and enough for community analysis (Additional file 1: Figure S1). After clustering at 97 % sequence identity, 13,911 OTUs were identified in this study, out of 120 OTUs were classified as archaea. Within the bacterial domain, 4 major microbial phyla accounted for 47.33 % − 59.67 % of all reads (Additional file 1: Figure S2), and they were *Proteobacteria* (18.05 % − 28.86 %), *Acidobacteria* (6.05 %, 23.44 %), *Actinobacteria* (5.65 %, 11.28 %) and *Chloroflexi* (3.99 %, 15.44 %). And about 14 % − 22 % of sequences were not assigned to any known phylum (Additional file 1: Figure S2). Microbial communities were more diverse at the genus level. The top five predominant microbial genera were *Acidobacteria_Gp6* (0.44 % − 11.83 %), *Ktedonobacter* (0.28 % − 8.88 %), *Spartobacteria_genera_incertae_sedis* (1.04 % − 4.99 %), *Acidobacteria_Gp1* (0.62 % − 6.82 %) and *Gemmatimonas* (0.89 % − 3.08 %). But the most abundant genus was different in each group.

**Fig. 1:**
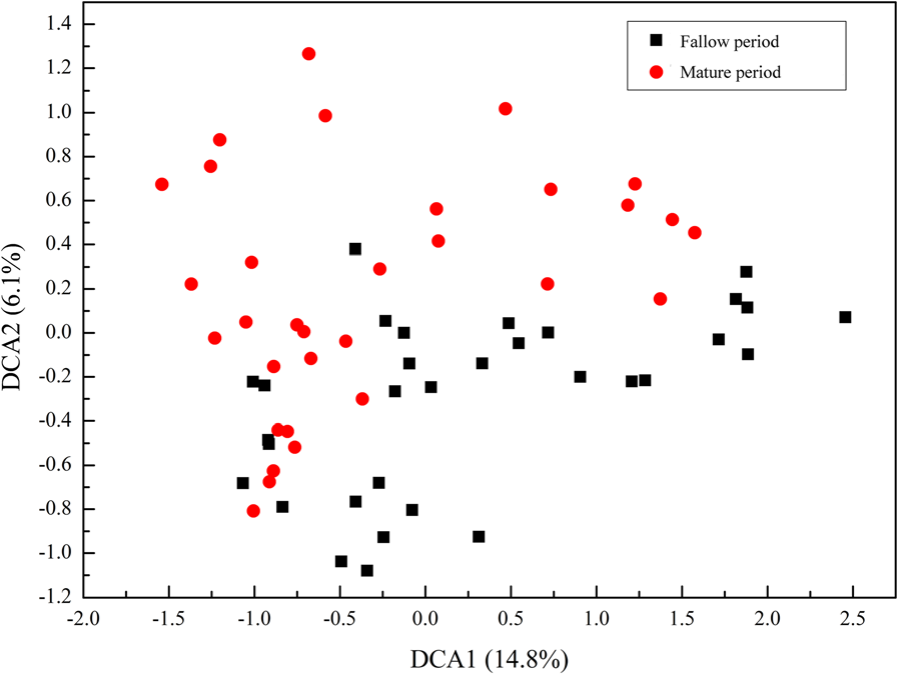
Detrended correspondence analysis (DCA) of 16r RNA gene sequencing data for all 80 samples. The values of DCA1 and DCA2 are percentages of total variations that can be attributed to the corresponding axis

To evaluate the similarity of these microbial communities in structure, we conducted dissimilarity test and DCA. DCA graph showed that samples in fallow period were separated clearly from tobacco mature period, indicating that soil microbial communities shifted during tobacco cultivation (Fig. 1). Dissimilarity test showed that microbial community composition and structure of 4 crop rotation systems were significantly (*p* < 0.01) different from each other, no matter in fallow period or in mature period (Tables 1 and 2). Both the Shannon diversity and Pielou evenness indices decreased significantly (*p* < 0.05) in mature period, except for MR. For example, in LR the diversity index decreased from 6.26 to 5.50 and evenness index decreased from 0.82 to 0.76 (Table 3).

**Table 1:** Dissimilarity test of 16S rRNA gene sequencing data among four groups in fallow period and mature period respectively

Sampling time	Group	Control		MR		LR	
		D	*P*	D	*P*	D	*P*
Fallow period	MR	0.48	<0.01				
	LR	0.51	<0.01	0.50	<0.01		
	TR	0.52	<0.01	0.50	<0.01	0.54	<0.01
Mature period	MR	0.48	<0.01				
	LR	0.46	<0.01	0.47	<0.01		
	TR	0.46	<0.01	0.47	<0.01	0.47	<0.01

*D* bray-cutis distance, *P* significance level

**Table 2:** Shannon diversity and Pielou evenness of soil microbial communities

Index	Sampling time	Control	MR	LR	TR
Shannon diversity	Fallow period	6.42^a^	6.36^a^	6.26^a^	6.43^a^
	Mature period	6.08^a^	6.50^b^	5.52^c^	5.91^ac^
	*P* value	**<0.05**	0.11	**<0.05**	**<0.05**
Pielou evenness	Fallow period	0.83^a^	0.81^b^	0.82^ab^	0.83^a^
	Mature period	0.81^ab^	0.82^a^	0.77^c^	0.79^b^
	*P* value	**<0.05**	0.23	**<0.05**	**<0.05**

Significant differences (*p* < 0.05) between two periods are indicated in bold. And significant (*p* < 0.05) differences among four groups are labeled with alphabet

### Microbial communities shift from fallow to tobacco mature period

**Fig. 2:**
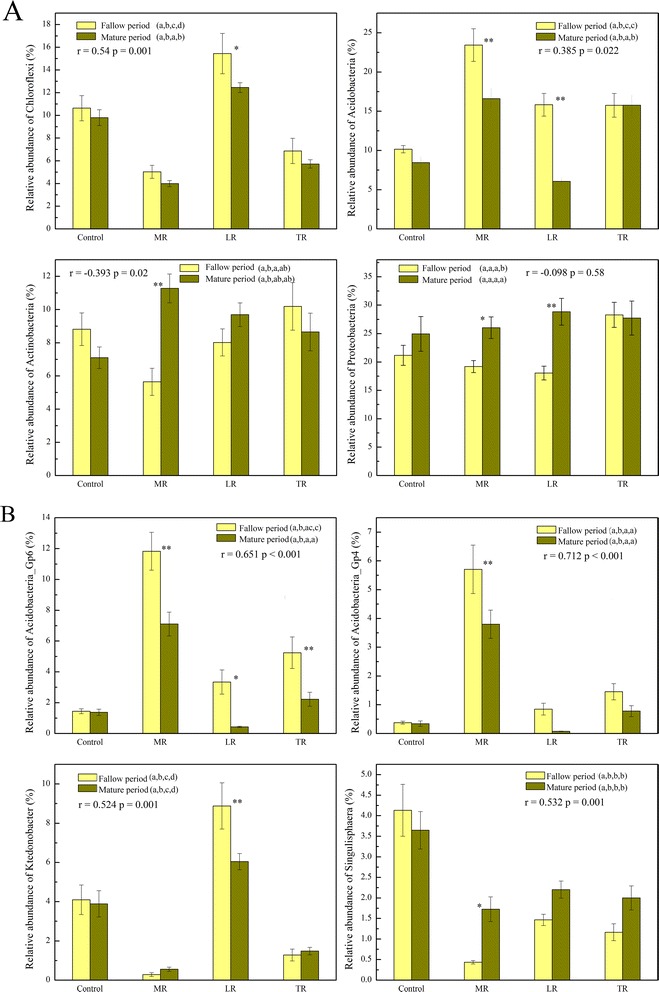
Relative abundances of dominant phyla (**a**) and genera (**b**) of soil microbial communities in 8 groups. Correlations in abundance of microbial populations between 2 periods are indicated with r and p values. Significant differences (*p* < 0.05) among 4 groups are indicated with alphabet. Significant differences between 2 periods are labeled with * (*p < 0.05, **p < 0.01)

Microbial community composition and structure were different in 4 rotation systems, such as *Chloroflexi* was less abundance in MR than in Control, and *Acidobacteria* was more abundant in TR than in Control (Fig. 2a). At the genus level, *Acidobacteria_Gp6* and *Acidobacteria_Gp4* were more abundant in MR while *Ktedonobacter* and *Singulisphaera* were more abundant in Control (Fig. 2b). However, most of the microbial populations had the similar abundance pattern in two periods. Pearson correlation analyses were conducted to evaluate the similarity in abundance pattern. Our results showed that relative abundances of *Acidobacteria*, *Chloroflex*i, *Planctomycetes*, *Firmicutes*, *Nitrospira* and *BRC1* in fallow period were positively correlated with that in tobacco mature period. These phyla accounted for 33.73 % − 52.26 % of total population except for unclassified OTUs, but accounted for 28.33 % − 42.67 % of total population including unclassified OTUs. Only the abundances of *Actinobacteria* showed negative correlation between 2 periods (Additional file 1: Table S2a). At the genus level, we found that 11 bacterial genera showed positive correlation in abundances between 2 periods, such as *Ktedonobacter*, *Singulisphaera* and *Acidobacteria_Gp2* (Additional file 1: Table S2b). And no microbial genus showed negative correlation between 2 periods. In summary, direct correlations were found for a large percent of microbial populations, indicating that there was a general response pattern of soil microbial communities to tobacco cultivation across rotation types, and that soil microbial communities in mature period were shaped by the fallow microbial communities.

Actually, tobacco planting had certain impacts on soil microbial communities, with community composition and structure changed from fallow stage to tobacco mature stage. Generally, relative abundances of *Proteobacteria* and *Actinobacteria* showed a tendency of increase at tobacco mature stage. On the contrary, *Chloroflexi* and *Acidobacteria* were more abundant at fallow stage, especially in LR (Fig. 2a). Especially, *Actinobacteria* was more abundant in mature period than fallow period only in MR, but showed no significant difference between two periods in other three groups. At the genus level, *Acidobacteria_Gp6* and *Acidobacteria_Gp4* were more abundant at fallow stage, while the abundances of *Singulisphaera* were higher at mature stage (Fig. 2b).

### Co-occurrences among different microbial populations

To understand the co-occurrence pattern among different microbial populations in 8 groups of microbial communities, 16S rRNA gene sequencing data were used to construct pMENs by RMT-based network approach. Major topological properties of 8 empirical pMENs showed that, with the same threshold (0.950), there were a lot more nodes and links in ecological networks of MR than other 3 groups no matter at fallow stage or tobacco mature stage (Additional file 1: Table S3). More importantly, the numbers of nodes and links decreased in tobacco mature period for networks of control, LR and TR, except for that of MR. The degree distributions in all constructed pMENs well fitted the power law model as linear correlations changed from 0.779 to 0.907. For the average path distance, it decreased at mature stage in pMENs of all groups except for MR, suggesting that ecological networks might more closely connected at fallow stage (Additional file 1: Table S3). The same tendency was also seen from Additional file 1: Figure S3.

**Fig. 3:**
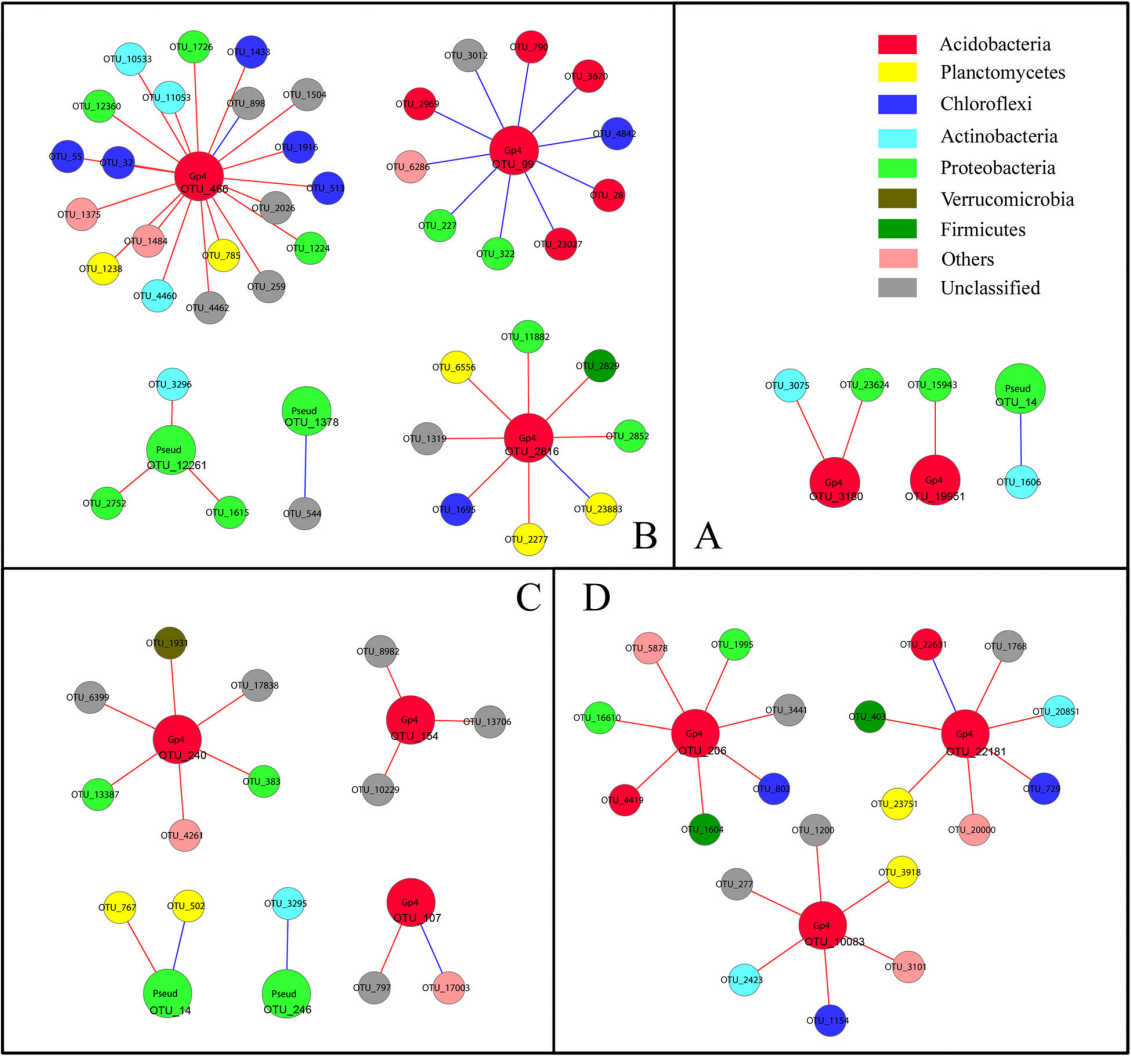
Network interactions of the only OTUs of *Gp4* and *Pseudomonas* in Control (**a**), MR (**b**), LR (**c**) and TR (**d**). Each node signifies an OTU which could correspond to a microbial population. Colors of the nodes indicate different major phylum. A blue line indicates a positive interaction between 2 individual nodes, while a red line indicates a negative interaction

To explore the mechanism of how (potential) probiotic bacteria interacted with other microbial populations to protect plants from disease, we analyzed the sub-networks of *Pseudomonas* and *Acidobacteria_Gp4*, whose abundances were negatively correlated to tobacco disease rate. Because we were interested in the co-occurrences relationships which were related to tobacco bacterial wilt directly, we only focused on the sub-networks of mature period when tobacco was severely diseased. Top 3 *Gp4* OTUs with the highest connections were chosen to construct the sub-network of each group. Most of the links (69 % − 95 %) were negative in all 4 sub-networks. Most OTUs of *Gp4* were negatively linked with OTUs of *Proteobacteria*, *Chloroflexi*, *Actinobacteria* and *Plantomycetes*. Of them, *Proteobacteria* and *Actinobacteria* had no significant correlation with tobacco disease rate, but the abundances of *Chloroflexi* and *Plantomycetes* were negatively correlated with tobacco disease rate. Of OTUs positively linked with *Gp4*, most of them were *Acidobacteria* and *Proteobacteria*, and *Acidobacteria* were potential probiotic bacteria. Compared with other three networks, *Gp4* in MR had more negative links with *Chloroflexi* and *Planctomycetes*, and more positive links with *Acidobacteria* (Fig. 3). It suggested that *Gp4* enhanced their cooperation with potential probiotic bacteria and competition with pathogens in MR.

### Relationship among soil properties, microbial communities and tobacco health

Tobacco disease was a key factor in shaping abundance patterns of many taxonomic groups. Based on Pearson correlation with tobacco, the abundances of 3 phyla were positively correlated with tobacco disease rate, including *Planctomycetes*, *Chloroflexi* and *Firmicutes*. On the contrary, abundances of 8 phyla were negatively correlated with tobacco morbidity (bacterial wilt), including *Acidobacteria*, *Verrucomicrobia*, *Crenarchaeota*, *Gemmatimonadetes*, *Bacteroidetes*, *Nitrospira*, *WS3*, and *BRC1* (Additional file 1: Table S4a). At the genus level, abundances of 7 genera (relative abundance > 0.5 %) decreased as the tobacco disease rate increased, whereas abundances of 13 genera increased with the increase of tobacco disease rate (Additional file 1: Table S4b). Especially, we found that the abundance of *Pseudomonas* was negatively correlated with incidence of tobacco bacterial wilt in tobacco mature period.

To reveal the relationship between microbial communities and soil properties, mantel test was conducted. Results showed that soil water content, pH and the content of K, Ca, and Mn had significant impacts on relative abundances of some microbial phyla and genera (Additional file 1: Table S5). For example, water content was correlated with the abundances of *Acidobacteria*, *Actinobacteria*, *Firmicutes* and *Nitrospira*. And soil Ca content had significant (*p* < 0.05) impacts on *Acidobacteria_Gp4*, *Acidobacteria_Gp6* and *Acidobacteria_Gp7*. Furthermore, Person correlation analysis showed that the impacts were positive, and both the amount of Ca and relative abundances of these 3 genera were negatively correlated with tobacco disease rate.

**Fig. 4:**
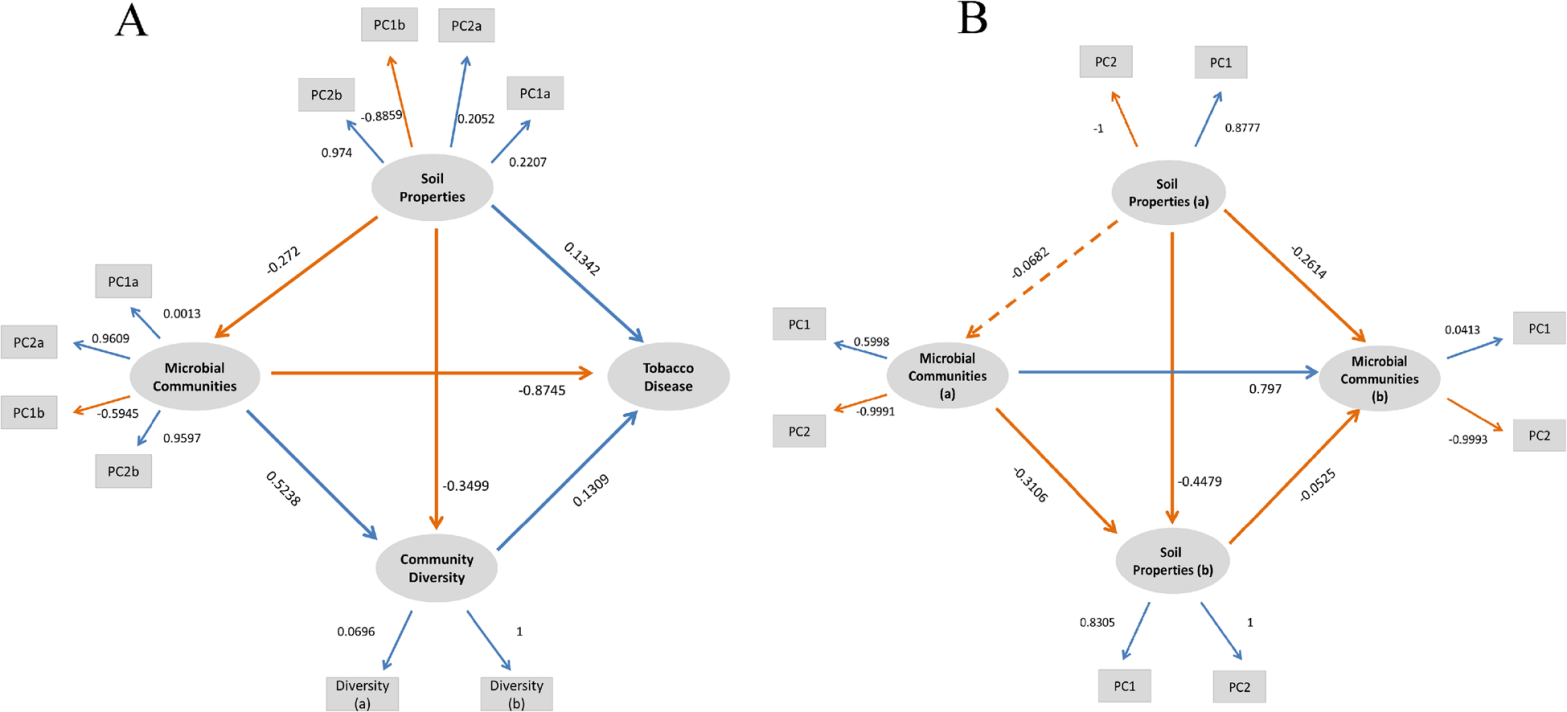
Partial least squares path modeling (PLSPM) about correlations among soil properties, microbial communities, community diversity and tobacco bacterial wilt (**a**), as well as among soil properties, microbial communities of fallow period and tobacco mature period (**b**)

Collectively, 2 PLSPMs were constructed to profile the relationship among soil properties, microbial communities and tobacco bacterial wilt. In the first model, the relationship among soil properties, microbial community structure, diversity and tobacco disease were explored. Results showed that soil properties had impacts on soil microbial communities; both soil properties and microbial communities influenced community diversity; and all of them contributed to tobacco bacterial wilt. All the correlations were significant (*p* < 0.01) in the model. Goodness of fit (Gof) value was 0.4599, bigger than 0.35, indicating that the model was reliable. In order to further reveal the relationship between microbial communities of fallow period and tobacco mature period, the second model was constructed. We found that soil properties in mature period were determined by soil properties and microorganisms of fallow period. And all of them shaped microbial communities of tobacco mature period (Fig. 4). Gof was 0.4995 and all correlations were significant (*p* < 0.01), except for the link between soil properties and microbial communities of fallow period.

## Discussion

Because of its value in microbial ecology and disease control of agriculture, the interaction mechanism between crop planting and soil microbial communities is of high significance in scientific research. It has been reported that plants could offer a specific environment that was selective to soil microbial communities, filtering out specific populations [29, 30]. So a general response pattern of soil microbial communities to crop planting is expected to exist. By investigating soil microbial communities during tobacco cultivation across different rotation types, we aimed to reveal the general response pattern of soil microbial communities to tobacco cultivation and how they shaped tobacco health ultimately. Particularly, this study suggested that interactions among microbial populations also played an important role in shaping crop health.

### The succession of soil microbial communities during tobacco cultivation

Soil microbial communities of tobacco mature period were shaped by that of fallow period. It was evidenced by that, most dominant microbial phyla and genera exhibited positive correlations in abundance between two periods, and no microbial genus showed negative correlation. In other word, the abundance pattern of major microbial populations in 4 groups remained the same, although their abundances changed with time. For instance, *Acidobacteria* was significantly more abundant in MR and less abundant in Control at fallow stage. Their relative abundances decreased at mature stage in almost all groups, but still remained more abundant in MR and less abundant in Control. In this way, we speculated that soil microbial community structure of tobacco mature stage ware mainly shaped by that of fallow stage. The result was conflict with some previous researches, which indicated that contribution of initial community to later community is usually small due to stochastic factors or changing of environmental factors [31, 32]. Probably because of the specific selection of plants to communities (e.g., root exudates) [29], stochastic or environmental factors have a relatively smaller impact on succession of soil microbial communities, resulting in the significant correlation between initial and later microbial composition in this study.

Actually, plants did have certain selective pattern on soil microbes, as previous study showed that bacterial communities not only adapt to plant type, but also change over time with the same plant type [33]. Here, the abundances of *Proteobacteria* and *Planctomycetes* increased while *Acidobacteria* and *Verrucomicrobia* decreased during tobacco cultivation. It might be related to root exudates (e.g., organic acids, lactam, esters, glycerol and nicotine) also [29, 34], which needs to be explored further. The impacts of root exudates on plant health related microorganisms are particularly significant. It was reported that plant root exudates could increase microbial activity in the rhizosphere, but some root exudates (e.g., phenylpropanoid) play important role in defense against non-host pathogens [34]. *Pseudomonas* could produce antifungal phloroglucinols in soils naturally suppressive to tobacco disease [35]. In this study, the abundance of *Pseudomonas* decreased in tobacco disease period while *Ralstonia* had no significant change in abundance between 2 periods. The result probably resulted from the different impact of tobacco root exudates on pathogens and antagonists.

Interestingly, many (not all) microbial populations which significantly correlated to tobacco disease rate, had abundance patterns shaped by that of previous period. It indicated that the abundance patterns of these potential disease inducible or suppressive species were already formed in fallow period. For instance, the relative abundance of *Chloroflexi* (mainly *Ktedonobacter*) was positively correlated with tobacco disease rate. They could be potential disease inducible microorganisms, because few plant-beneficial properties were associated with *Chloroflexi.* For example, they can’t fix nitrogen, thus may compete for nitrogen resource with tobacco plants [36]. On the contrary, *Acidobacteria* (including *Acidobacteria_Gp6* and *Acidobacteria_Gp4*) were negatively correlated with tobacco disease, suggesting their possible function in disease suppression. They had genes that encode polyketide synthase and nonribosomal peptide synthase enzymes, which well known for their roles in the synthesis of antibiotics and antifungals [18, 37]. These characteristics made most of *Acidobacteria* populations beneficial to tobacco health. However, both of them had abundance pattern shaped at fallow stage, suggesting the significance of initial soil microbial communities before tobacco transplanting to tobacco health.

### The impacts of crop rotation on microbial succession and tobacco health

One of the major results of this study was that microbial community diversity decreased dramatically after tobacco planting but still remained relatively high in MR, which was of low tobacco disease rate. A previous study showed that microbial diversity index showed a trend of decreasing under continuous tobacco cropping [38], indicating a negative influence of tobacco on soil microbial diversity. More importantly, rotation may affect microbial community diversity reversely. In some cases, Shannon diversity under rotation cropping was significantly higher than that in continuous cropping [18, 39]. So we supposed that the maize rotation may decrease the incidence of tobacco bacterial wilt by alleviating the decrease of diversity during tobacco plantation. In other words, soil microbial communities with high diversity may have advantages in preventing plant from disease. Griffiths reported that there was no direct relationship between biodiversity and function, but the soils with the highest biodiversity were more resistant to the stress than soils with impaired biodiversity [40]. And there is evidence that soil microbial diversity confers protection against soil-borne disease, significant for agricultural sustainability [35]. Collectively, our results supported that tobacco farmlands with high biodiversity were more resistant to infection of pathogen.

Decreasing the co-occurrences/connections among microbes might be another general pattern of how soil microbial communities responded to tobacco planting. In this study, the number of nodes and links in pMENs of all groups decreased in tobacco mature period except for MR. A study suggested a positive correlation between biodiversity and interactions of communities, as elevated biodiversity was observed in more connected communities [41]. Consistent with the previous study, MR microbial communities had higher diversity and larger ecological network. There were extensive cooperation and competition among different populations, and the maintaining of such relationships makes community a homeostatic systems [42]. If the balance is destroyed by environments or invader, certain species of the community will prosper or decline even extinct [43]. In this study the decrease of co-occurrence among microbial populations might result from/in prosper of certain species, destroyed biodiversity and destruction of balance. Therefore, the impaired interactions among microbial populations might make communities more susceptible to invasion of pathogens.

Further analyses indicated that there was stronger competition between potential disease suppressive (e.g., *Acidobacteria*) and inducible bacteria (e.g., *Chloroflexi*) in maize rotation systems. Previous studies showed that some bacteria could protect the plant directly by producing antibacterial agent, or indirectly by enhancing rhizosphere function of antagonistic populations [8]. Here, *Acidobacteria_Gp4* had more negative links with disease inducible bacteria (e.g., *Chloroflexi* and *Planctomycetes*), and more positive links with disease suppressive populations (e.g., *Acidobacteria*). It indicated that *Gp4* enhanced their cooperation with probiotic bacteria and competition with potential pathogens in MR. Pathogenesis is a process in which pathogens and biocontrol agents compete with each other, supporting our results [4]. The enhanced interactions limited the prosperity of *Raltonia* and other potential pathogens, making maize rotation an effective system in controlling tobacco bacterial wilt. In conclusion, our results indicated that plant disease resulted largely from the interactions among different microbial populations, particularly between pathogens and probiotic bacteria, although further experimental verifications are necessary.

Finally, we conducted PLSPM analyses to profile the complex interactions among soil properties, microbial communities and tobacco health. On the one hand, soil properties could affect tobacco directly. For example, Ca is not only a necessary element for plant growth, it could also increase tobacco’s resistance to pathogen indirectly. Moshe Sagi found that the plant homolog can be stimulated directly by Ca_2_^+^ to produce O_2_^-^, which was considered to be a component of the resistance response of plants to pathogen challenge [44]. It supported our result that the amount of Ca in soil was negatively correlated with tobacco morbidity. On the other hand, soil properties could affect tobacco health indirectly through soil microbial communities. Noah Fierer and Robert B. Jackson reported the diversity and richness of soil bacterial communities could largely be explained by soil pH [6]. In this study, soil pH (5.5 ~ 6.5) and water content (15 % ~ 25 %) were suitable for tobacco plantation. They were different in 4 rotation groups or between 2 periods, indicating their impacts on soil microbial communities, consistent with previous study. Then, soil microorganisms impact tobacco health in many ways. Some of the indigenous microorganisms protect susceptible crops from certain pathogens, whereas disease-conducive microorganisms infect plants or permit spread of the pathogens [8, 45]. By studying the shift of soil microorganisms in composition, structure and co-occurrence during tobacco planting across different rotation types, our research profiled a general picture of the succession pattern of microbial communities and its relationship with tobacco health.

## Conclusions

In summary, we found that (i) both soil microbial communities of fallow stage and tobacco selection shaped the communities of tobacco mature stage; (ii) effective rotation crop (maize) could decrease the incidence of tobacco bacterial wilt by alleviating the decrease in diversity and co-occurrences of microbial populations.

## Additional file

Additional file 1
**Figure S1**. Rarefaction curves of 16r RNA gene sequencing data. **Figure S2**. Composition and structure of soil microbial communities in each group. **Figure S3**. Phylogenetic molecular ecological networks (pMEN) of microbial communities in each group, and number of nodes and links of each pMEN. **Table S1**. Soil properties. **Table S2(a)**. Correlation of microbial populations in abundance between two periods at the phylum level. (b). Correlation of microbial populations in abundance between two periods at the genus level. **Table S3**. Topological properties of the empirical pMENs of microbial communities in eight groups. **Table S4(a)**. Correlation between abundance of microbial populations and tobacco morbidity at the phylum level. (b). Correlation between abundance of microbial populations and tobacco morbidity at the genus level. **Table S5(a)**. Mantel test of sequencing data with environmental attributes at the phylum level. (b). Mantel test of sequencing data with environmental attributes at the genus level. (DOCX 1100 kb)
