# Supplementary material for: The succession pattern of soil microbial communities and its relationship with tobacco bacterial wilt
Source: BMC Microbiol. 2016 Oct 6;16:233. doi: 10.1186/s12866-016-0845-x (PMC5054579; doi:10.1186/s12866-016-0845-x)
Supplement: Additional file 1 — Figure S1. Rarefaction curves of 16r RNA gene sequencing data. Figure S2. Composition and structure of soil microbial communities in each group. Figure S3. Phylogenetic molecular ecological networks (pMEN) of microbial communities in each group, and number of nodes and links of each pMEN. Table S1. Soil properties. Table S2(a). Correlation of microbial populations in abundance between two periods at the phylum level. (b). Correlation of microbial populations in abundance between two periods at the genus level. Table S3. Topological properties of the empirical pMENs of microbial communities in eight groups. Table S4(a). Correlation between abundance of microbial populations and tobacco morbidity at the phylum level. (b). Correlation between abundance of microbial populations and tobacco morbidity at the genus level. Table S5(a). Mantel test of sequencing data with environmental attributes at the phylum level. (b). Mantel test of sequencing data with environmental attributes at the genus level. (DOCX 1100 kb) [file 12866_2016_845_MOESM1_ESM.docx]

## The Succession Pattern of Soil Microbial Communities and Its Relationship with Tobacco Bacterial Wilt (Supplementary)

Jiaojiao Niu^1,2,+^, Zhongwen Rang^3+^, Chao Zhang^3^, Wu Chen^3^, Feng Tian^4^, Huaqun Yin^1,2^*& Linjian Dai^3^*

^1^School of Minerals Processing and Bioengineering, Central South University, Changsha 410083, China, ^2^Key laboratory of Biometallurgy, Ministry of Education, Changsha 410083, China, ^3^ College of agronomy, Hunan Agricultural University, Changsha 410128, China, ^4^Tobacco monopoly bureau of Xiangxi Autonomous Prefecture, Hunan Jishou 416000, China.

Authors:

Jiaojiao Niu ([jjniu15@hotmail.com](mailto:jjniu15@hotmail.com))

Zhongwen Rang (rzwronger@126.com)

Chao Zhang (chaozhang91@126.com)

Wu Chen ([chenwuwarrior@163.com](mailto:chenwuwarrior@163.com))

Feng Tian ([hnxxtianf@163.com](mailto:hnxxtianf@163.com))

Huaqun Yin (yinhuaqun@gmail.com)

Linjian Dai (linjianpaper@foxmail.com)

*Corresponding author E-mail: yinhuaqun@gmail.com; [linjianpaper@foxmail.com](mailto:linjianpaper@foxmail.com)

**^+^**These authors contributed equally to this work.


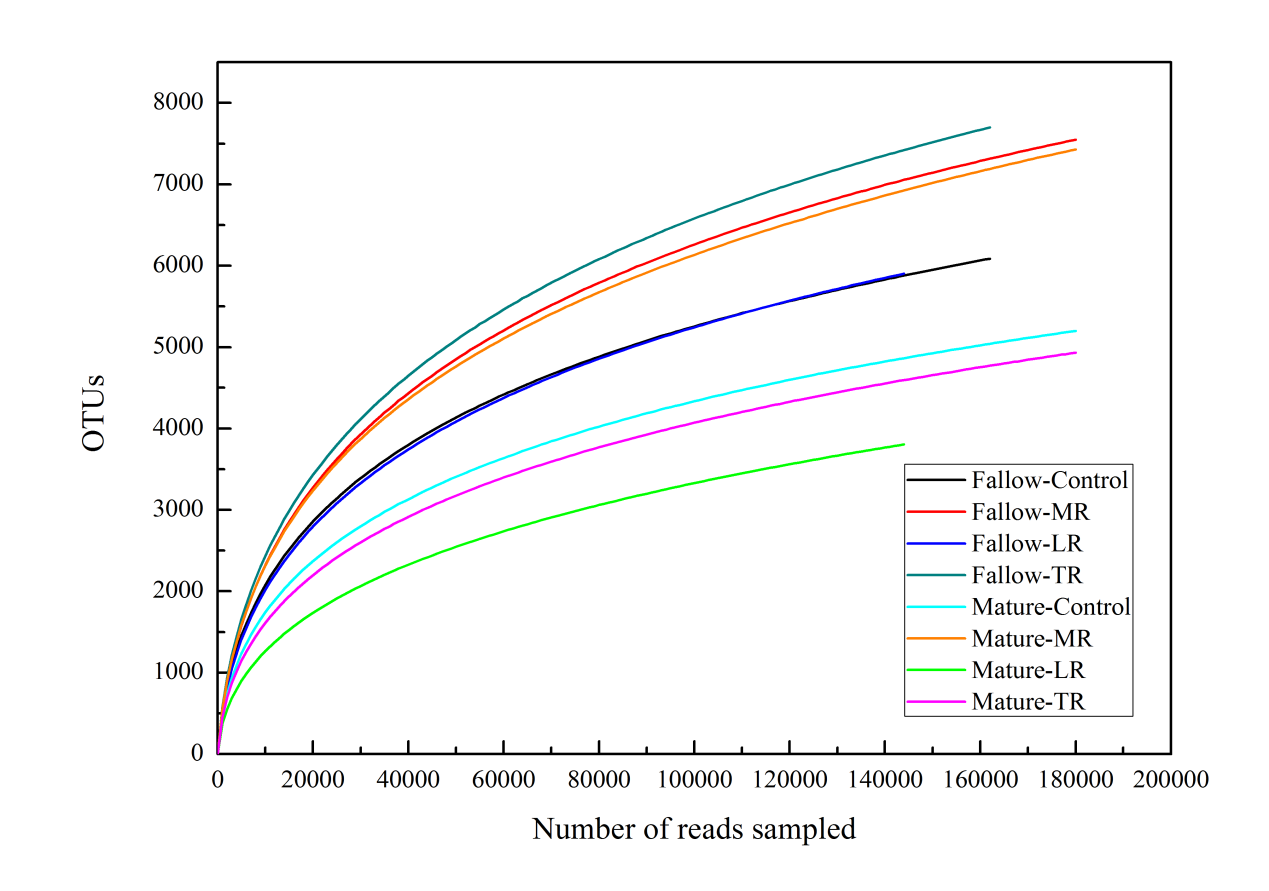


Figure S1. Rarefaction curves of 16r RNA gene sequencing data.


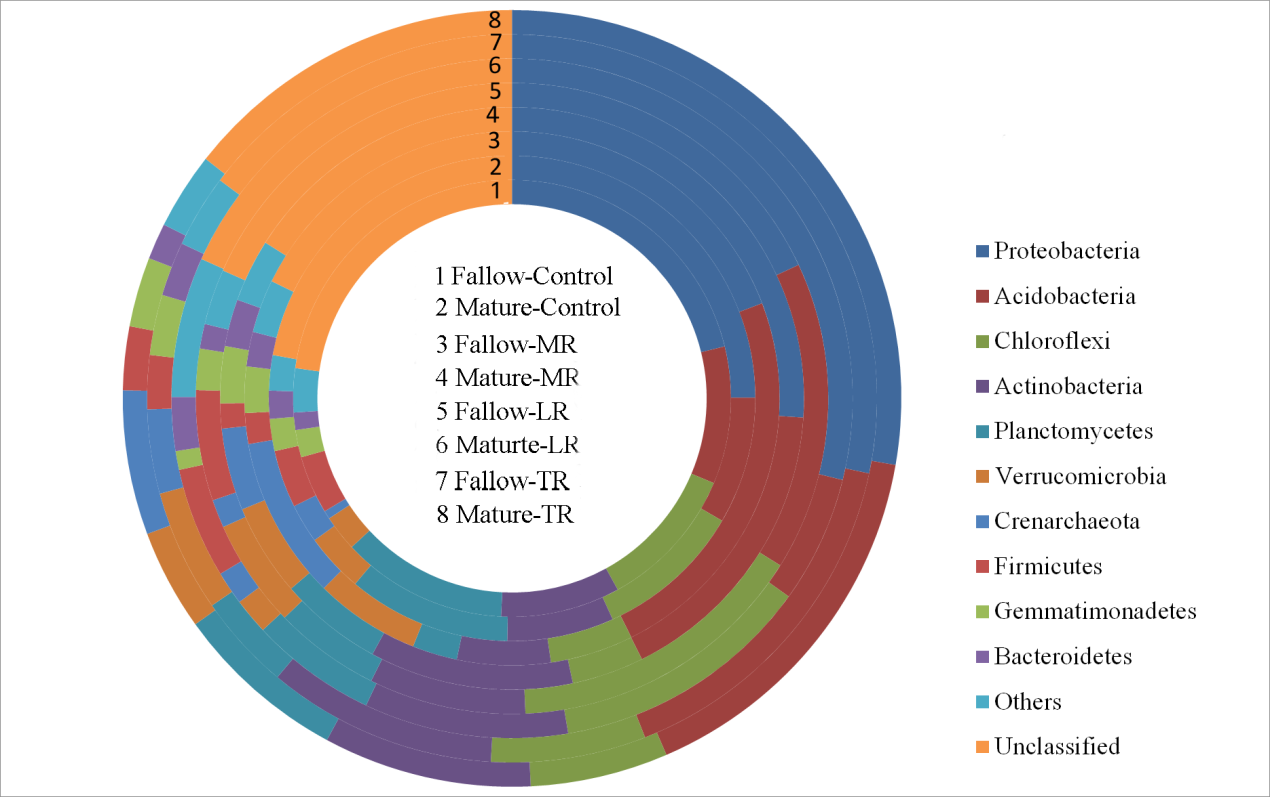


Figure S2. Composition and structure of soil microbial communities in each group.


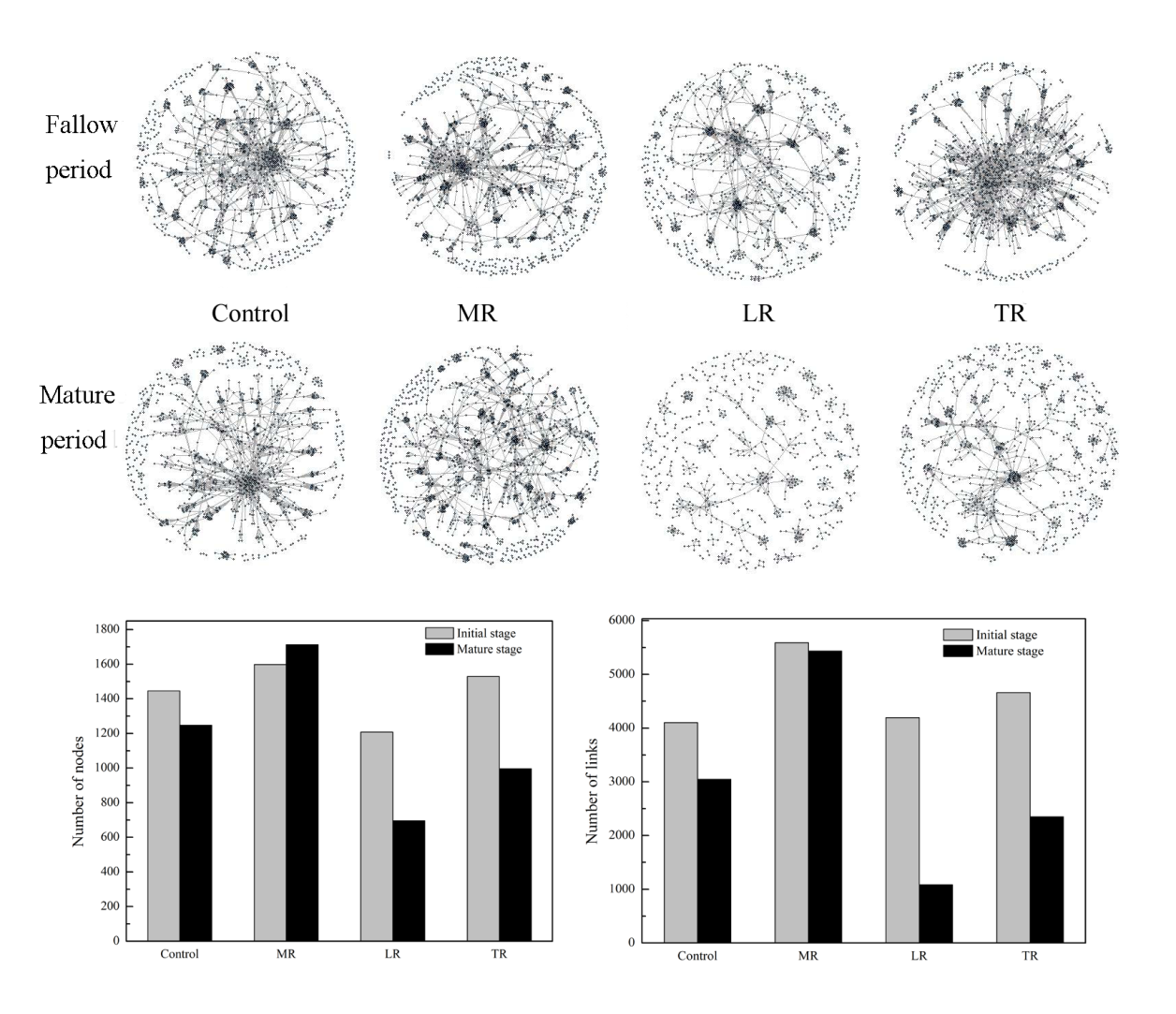


Figure S3. Phylogenetic molecular ecological networks (pMEN) of microbial communities in each group, and number of nodes and links of each pMEN.

Table S1. Soil properties.

|  | Group | Water content (%) | PH | K | Ca | Mn | Fe | Cr | Co | Ni |
| --- | --- | --- | --- | --- | --- | --- | --- | --- | --- | --- |
| Fallow period | Control | 29.08±3.44 (a) | 5.31±0.44 (a) | 10848.22±562.88 (a) | 902.78±116.037 (ab) | 1296.33±121.57 (a） | 30662.44±3065.78 (a) | 49.44±11.66 (a) | 10.00±1.65 (a) | 35.67±15.70 (a) |
|  | MR | 22.34±0.65 (b) | 4.79±0.21 (b) | 12945.80±937.59 (b) | 1780.70±911.14 (c) | 1377.20±132.44 (a) | 38402.90±7918.57 (a) | 66.50±25.48 (ab) | 12.48±4.01 (a) | 50.50±13.12 (b) |
|  | LR | 25.24±1.90 (ab) | 4.29±0.25 (c) | 12334.00±1097.87 (b) | 870.37±296.57 (a) | 1098.37±333.72 (a) | 55809.37±15117.65 (b) | 83.87±20.69 (b) | 22.00±6.85 (b) | 67.62±17.58 (bc) |
|  | TR | 26.44±0.75 (ab) | 4.66±0.34 (b) | 11977.55±1158.70 (b) | 1221.33±248.50 (b) | 1671.55±419.54 (b) | 55596.44±21216.93 (b) | 88.33±27.33 (b) | 20.40±7.98 (b) | 69.33±30.11 (c) |
| Mature period | Control | 15.52±0.64 (a) | 5.40±0.49 (a) | 11881.50±1788.65 (a) | 976.80±320.26 (ab) | 1094.90±210.59 (a) | 20297.10±4229.78 (a) | 33.90±34.28 (a) | 7.48±2.42 (a) | 9.90±15.31 (a) |
|  | MR | 15.99±0.99 (a) | 4.79±0.21 (b) | 14300.30±2422.71 (b) | 1551.50±235.51 (c) | 1317.70±260.78 (a) | 28581.90±5767.71 (a) | 42.60±37.02 (ab) | 9.41±3.82 (a) | 14.00±21.92 (b) |
|  | LR | 15.74±1.32 (a) | 4.43±0.23 (c) | 14353.13±2239.09 (b) | 864.63±175.88 (a) | 860.50±124.06 (a) | 31889.50±8031.42 (b) | 65.87±38.92 (b) | 11.30±3.44 (b) | 22.50±23.55 (bc) |
|  | TR | 15.85±0.70 (a) | 4.62±0.34 (b) | 14822.50±1085.24 (b) | 1130.00±312.34 (b) | 2503.20±2089.21 (b) | 32352.60±9283.63 (b) | 62.30±28.38 (b) | 10.22±5.64 (b) | 37.20±31.24 (c) |
|  | p value | **< 0.001** | 0.654 | **< 0.001** | 0.547 | 0.631 | **< 0.001** | **0.007** | **< 0.001** | **< 0.001** |

*All values are in ppm except for pH and water content. Significant differences (p < 0.05) between two periods are indicated in bold. And significant differences (p < 0.05) among four groups are labeled with alphabet.

Table S2(a). Correlation of microbial populations in abundance between two periods at the phylum level.

| Phylum | r | p |
| --- | --- | --- |
| *Proteobacteria* | -0.097 | 0.58 |
| *Planctomycetes* | **0.496** | **0.002** |
| *Chloroflexi* | **0.54** | **0.001** |
| *Firmicutes* | **0.428** | **0.01** |
| *Acidobacteria* | **0.385** | **0.022** |
| *Verrucomicrobia* | 0.063 | 0.72 |
| *Crenarchaeota* | 0.167 | 0.338 |
| *Actinobacteria* | **-0.393** | **0.02** |
| *Gemmatimonadetes* | 0.209 | 0.229 |
| *Bacteroidetes* | -0.073 | 0.675 |
| *Armatimonadetes* | 0.147 | 0.398 |
| *Cyanobacteria* | -0.211 | 0.224 |
| *TM7* | 0.268 | 0.119 |
| *Nitrospira* | **0.361** | **0.033** |
| *WS3* | 0.052 | 0.767 |
| *Euryarchaeota* | -0.209 | 0.229 |
| *Chlamydiae* | -0.075 | 0.668 |
| *OD1* | 0.296 | 0.084 |
| *BRC1* | **0.497** | **0.002** |
| *Spirochaetes* | 0.152 | 0.382 |
| *Deinococcus-Thermus* | 0.284 | 0.099 |

Table S2(b). Correlation of microbial populations in abundance between two periods at the genus level.

| Genus | r | p |
| --- | --- | --- |
| *Acidobacteria_Gp6* | **0.651** | **0** |
| *Acidobacteria_Gp4* | **0.712** | **0** |
| *Acidobacteria_Gp7* | **0.585** | **0** |
| *Spartobacteria_genera_incertae_sedis* | 0.062 | 0.724 |
| *Acidobacteria_Gp1* | 0.265 | 0.124 |
| *Acidobacteria_Gp2* | **0.387** | **0.022** |
| *Singulisphaera* | **0.532** | **0.001** |
| *Planctomyces* | 0.122 | 0.487 |
| *Ktedonobacter* | **0.524** | **0.001** |
| *Gemmatimonas* | 0.209 | 0.229 |
| *Sphingosinicella* | 0.328 | 0.054 |
| *Dokdonella* | 0.123 | 0.483 |
| *Sphingomonas* | 0.079 | 0.65 |
| *Rhodanobacter* | 0.029 | 0.869 |
| *Subdivision3_genera_incertae_sedis* | **0.364** | **0.032** |
| *Acidobacteria_Gp16* | 0.304 | 0.076 |
| *Arthrobacter* | -0.138 | 0.429 |
| *Gemmata* | **0.383** | **0.023** |
| *Streptophyta* | -0.178 | 0.306 |
| *Bradyrhizobium* | 0.251 | 0.145 |
| *Blastopirellula* | 0.212 | 0.22 |
| *Acidobacteria_Gp3* | 0.088 | 0.616 |
| *Massilia* | -0.325 | 0.057 |
| *TM7_genera_incertae_sedis* | 0.268 | 0.119 |
| *Pseudolabrys* | 0.203 | 0.242 |
| *Conexibacter* | 0.166 | 0.341 |
| *Burkholderia* | **0.461** | **0.005** |
| *Zavarzinella* | 0.079 | 0.653 |
| *WS3_genera_incertae_sedis* | 0.052 | 0.767 |
| *Skermanella* | 0.093 | 0.597 |

*Significant correlations (p < 0.01) are indicated in bold.

Table S3. Topological properties of the empirical pMENs of microbial communities in eight groups.

|  | Community | No.of original OTUs | Similarity threshold | Total nodes | Total links | R square of power-law | Average degree (avgK) | Average clustering coefficient (avgCC) | Average path distance (GD) |
| --- | --- | --- | --- | --- | --- | --- | --- | --- | --- |
| Fallow period | Control | 2308 | 0.95 | 1446 | 4104 | 0.876 | 5.676 | 0.153 | 5.693 |
|  | MR | 2304 | 0.95 | 1598 | 5588 | 0.827 | 6.994 | 0.15 | 5.221 |
|  | LR | 1761 | 0.95 | 1208 | 4194 | 0.838 | 6.944 | 0.122 | 4.691 |
|  | TR | 1964 | 0.95 | 1529 | 4660 | 0.852 | 6.095 | 0.149 | 4.898 |
| Mature period | Control | 1746 | 0.95 | 1247 | 3048 | 0.893 | 4.889 | 0.157 | 4.203 |
|  | MR | 2441 | 0.95 | 1713 | 5433 | 0.779 | 6.343 | 0.107 | 6.075 |
|  | LR | 1239 | 0.95 | 696 | 1086 | 0.84 | 3.121 | 0.131 | 0.705 |
|  | TR | 1590 | 0.95 | 996 | 2349 | 0.907 | 4.717 | 0.123 | 4.36 |

Table S4(a). Correlation between abundance of microbial populations and tobacco morbidity at the phylum level.

| Phylum | r | p |
| --- | --- | --- |
| *Proteobacteria* | 0.022 | 0.895 |
| *Planctomycetes* | **0.420** | **0** |
| *Chloroflexi* | **0.360** | **0.002** |
| *Firmicutes* | **0.372** | **0.001** |
| *Acidobacteria* | **-0.480** | **0** |
| *Verrucomicrobia* | **-0.319** | **0.006** |
| *Crenarchaeota* | **-0.366** | **0.001** |
| *Actinobacteria* | 0.031 | 0.795 |
| *Gemmatimonadetes* | **-0.249** | **0.032** |
| *Bacteroidetes* | **-0.240** | **0.039** |
| *Armatimonadetes* | -0.095 | 0.419 |
| *Cyanobacteria/Chloroplast* | 0.059 | 0.617 |
| *TM7* | 0.116 | 0.324 |
| *Nitrospira* | **-0.366** | **0.001** |
| *WS3* | **-0.235** | **0.044** |
| *Euryarchaeota* | -0.111 | 0.348 |
| *Chlamydiae* | 0.156 | 0.184 |
| *OD1* | -0.187 | 0.111 |
| *BRC1* | **-0.598** | **0** |
| *Spirochaetes* | 0.003 | 0.978 |
| *Deinococcus-Thermus* | 0.131 | 0.264 |

Table S4(b). Correlation between abundance of microbial populations and tobacco morbidity at the genus level.

| Genus | r | p |
| --- | --- | --- |
| *Acidobacteria_Gp6* | **-0.687** | **0** |
| *Ktedonobacter* | **0.320** | **0.005** |
| *Spartobacteria_genera_incertae_sedis* | **-0.401** | **0** |
| *Acidobacteria_Gp1* | **0.542** | **0** |
| *Gemmatimonas* | **-0.249** | **0.032** |
| *Sphingosinicella* | 0.132 | 0.264 |
| *Singulisphaera* | **0.413** | **0** |
| *Acidobacteria_Gp4* | **-0.753** | **0** |
| *Dokdonella* | 0.154 | 0.19 |
| *Sphingomonas* | 0.179 | 0.127 |
| *Acidobacteria_Gp2* | **0.472** | **0** |
| *Rhodanobacter* | 0.072 | 0.541 |
| *Subdivision3_genera_incertae_sedis* | 0.121 | 0.305 |
| *Acidobacteria_Gp16* | **-0.491** | **0** |
| *Planctomyces* | **0.485** | **0** |
| *Arthrobacter* | -0.102 | 0.388 |
| *Gemmata* | **0.276** | **0.017** |
| *Streptophyta* | 0.054 | 0.65 |
| *Bradyrhizobium* | **0.338** | **0.003** |
| *Blastopirellula* | **0.359** | **0.002** |
| *Acidobacteria_Gp3* | **0.382** | **0.001** |
| *Massilia* | 0.201 | 0.086 |
| *TM7_genera_incertae_sedis* | 0.116 | 0.324 |
| *Acidobacteria_Gp7* | **-0.602** | **0** |
| *Pseudolabrys* | **0.395** | **0** |
| *Conexibacter* | **0.467** | **0** |
| *Burkholderia* | **0.458** | **0** |
| *Zavarzinella* | -0.006 | 0.957 |
| *WS3_genera_incertae_sedis* | **-0.235** | **0.044** |
| *Skermanella* | **0.383** | **0.001** |

*Significant correlations (p < 0.05) are indicated in bold.

Table S5(a). Mantel test of sequencing data with environmental attributes at the phylum level.

|  | Water Content (%) | Tobacco disease rate (%) | pH | K | Ca | Cr | Mn | Co |
| --- | --- | --- | --- | --- | --- | --- | --- | --- |
| Whole | 0.281 | **0.001** | 0.813 | 0.202 | 0.157 | 0.138 | 0.071 | 0.7 |
| *Acidobacteria* | **0.038** | **0.001** | 0.556 | 0.98 | **0.001** | 0.887 | 0.7 | 0.575 |
| *Actinobacteria* | **0.041** | **0.005** | 0.944 | 0.155 | 0.489 | **0.031** | 0.076 | **0.047** |
| *Bacteroidetes* | 0.453 | 0.58 | 0.814 | **0.036** | 0.596 | **0.006** | 0.072 | 0.176 |
| *Chloroflexi* | 0.059 | 0.158 | 0.21 | 0.449 | 0.203 | 0.367 | 0.066 | 0.247 |
| *Crenarchaeota* | 0.917 | **0.001** | 0.954 | 0.899 | 0.139 | 0.218 | 0.642 | 0.815 |
| *Cyanobacteria* | 0.123 | 0.947 | 0.292 | 0.073 | 0.574 | 0.237 | 0.222 | 0.766 |
| *Firmicutes* | **0.005** | 0.915 | **0.026** | 0.656 | 0.323 | 0.666 | 0.583 | 0.874 |
| *Gemmatimonadetes* | 0.462 | **0.001** | 0.813 | 0.406 | **0.024** | 0.257 | **0.004** | **0.01** |
| *Nitrospira* | **0.031** | **0.001** | 0.479 | 0.059 | 0.102 | 0.879 | 0.979 | 0.887 |
| *Planctomycetes* | 0.109 | 0.194 | 0.096 | 0.619 | 0.186 | 0.572 | 0.697 | 0.964 |
| *Proteobacteria* | 0.13 | 0.788 | 0.402 | **0.011** | 0.898 | 0.255 | **0.014** | 0.371 |
| *TM7* | 0.347 | 0.217 | 0.833 | **0.048** | 0.708 | 0.104 | 0.541 | 0.816 |
| *Verrucomicrobia* | 0.851 | **0.001** | 0.896 | 0.984 | 0.079 | 0.07 | 0.234 | **0.029** |
| *WS3* | 0.076 | **0.013** | 0.195 | 0.979 | **0.018** | 0.997 | 0.91 | 0.66 |

Table S5(b). Mantel test of sequencing data with environmental attributes at the genus level.

|  | Tobacco disease rate (%) | Water content (%) | pH | Ca | Mn | Co | Ni |
| --- | --- | --- | --- | --- | --- | --- | --- |
| Whole | **0.028** | 0.337 | 0.566 | 0.055 | **0.02** | 0.161 | 0.395 |
| *Dokdonella* | 0.844 | 0.63 | 0.571 | 0.344 | **0.036** | 0.33 | 0.365 |
| *Gemmatimonas* | **0.001** | 0.464 | 0.79 | **0.032** | **0.001** | **0.024** | 0.311 |
| *Acidobacteria_Gp1* | **0.047** | 0.615 | 0.472 | 0.246 | 0.168 | 0.424 | 0.439 |
| *Acidobacteria_Gp2* | 0.816 | **0.001** | **0.019** | 0.34 | 0.785 | 0.877 | 0.906 |
| *Acidobacteria_Gp4* | **0.001** | 0.996 | 0.997 | **0.001** | 0.913 | 0.616 | 0.674 |
| *Acidobacteria_Gp6* | **0.001** | 0.787 | 0.955 | **0.001** | 0.642 | 0.157 | 0.218 |
| *Acidobacteria_Gp7* | **0.001** | 0.662 | 0.972 | **0.037** | 0.433 | **0.002** | **0.037** |
| *Ktedonobacter* | 0.372 | 0.086 | 0.228 | 0.145 | 0.362 | 0.332 | 0.408 |
| *Planctomyces* | **0.004** | 0.534 | 0.484 | 0.149 | 0.198 | 0.827 | 0.374 |
| *Singulisphaera* | 0.192 | **0.015** | **0.045** | 0.335 | 0.753 | 0.951 | 0.573 |
| *Spartobacteria* | **0.001** | 0.851 | 0.87 | 0.063 | 0.172 | 0.051 | 0.257 |
| *Sphingomonas* | **0.001** | 0.19 | 0.572 | 0.275 | **0.02** | **0.012** | **0.034** |
| *Sphingosinicella* | 0.489 | 0.508 | 0.633 | 0.688 | **0.001** | 0.071 | 0.377 |

*Significant impacts (p < 0.05) are indicated in bold.
